# Patient-reported outcomes for people with diabetes: what and how to measure? A narrative review

**DOI:** 10.1007/s00125-023-05926-3

**Published:** 2023-05-24

**Authors:** Caroline B. Terwee, Petra J. M. Elders, Marieke T. Blom, Joline W. Beulens, Olaf Rolandsson, Alize A. Rogge, Matthias Rose, Nicola Harman, Paula R. Williamson, Frans Pouwer, Lidwine B. Mokkink, Femke Rutters

**Affiliations:** 1 Amsterdam UMC, Department of Epidemiology and Data Science, Vrije Universiteit Amsterdam, Amsterdam, the Netherlands; 2 Amsterdam Public Health Research Institute, Methodology, Amsterdam, the Netherlands; 3 Amsterdam UMC, Department of General Practice, Vrije Universiteit Amsterdam, Amsterdam, the Netherlands; 4 Department of Public Health and Clinical Medicine, Family Medicine, Umeå University, Umeå, Sweden; 5 Center for Patient-Centered Outcomes Research, Department of Psychosomatic Medicine, Charité – Universitätsmedizin Berlin, Berlin, Germany; 6 Department of Health Data Science, University of Liverpool, Liverpool, UK; 7 Steno Diabetes Center Odense, Odense, Denmark; 8 Department of Psychology, University of Southern Denmark, Odense, Denmark; 9 Amsterdam UMC, Department of Medical Psychology, Vrije Universiteit Amsterdam, Amsterdam, the Netherlands

**Keywords:** Diabetes, Patient-reported outcome, Patient-reported outcome measure, Review, Standardisation

## Abstract

Patient-reported outcomes (PROs) are valuable for shared decision making and research. Patient-reported outcome measures (PROMs) are questionnaires used to measure PROs, such as health-related quality of life (HRQL). Although core outcome sets for trials and clinical practice have been developed separately, they, as well as other initiatives, recommend different PROs and PROMs. In research and clinical practice, different PROMs are used (some generic, some disease-specific), which measure many different things. This is a threat to the validity of research and clinical findings in the field of diabetes. In this narrative review, we aim to provide recommendations for the selection of relevant PROs and psychometrically sound PROMs for people with diabetes for use in clinical practice and research. Based on a general conceptual framework of PROs, we suggest that relevant PROs to measure in people with diabetes are: disease-specific symptoms (e.g. worries about hypoglycaemia and diabetes distress), general symptoms (e.g. fatigue and depression), functional status, general health perceptions and overall quality of life. Generic PROMs such as the 36-Item Short Form Health Survey (SF-36), WHO Disability Assessment Schedule (WHODAS 2.0), or Patient-Reported Outcomes Measurement Information System (PROMIS) measures could be considered to measure commonly relevant PROs, supplemented with disease-specific PROMs where needed. However, none of the existing diabetes-specific PROM scales has been sufficiently validated, although the Diabetes Symptom Self-Care Inventory (DSSCI) for measuring diabetes-specific symptoms and the Diabetes Distress Scale (DDS) and Problem Areas in Diabetes (PAID) for measuring distress showed sufficient content validity. Standardisation and use of relevant PROs and psychometrically sound PROMs can help inform people with diabetes about the expected course of disease and treatment, for shared decision making, to monitor outcomes and to improve healthcare. We recommend further validation studies of diabetes-specific PROMs that have sufficient content validity for measuring disease-specific symptoms and consider generic item banks developed based on item response theory for measuring commonly relevant PROs.

**Graphical Abstract:**

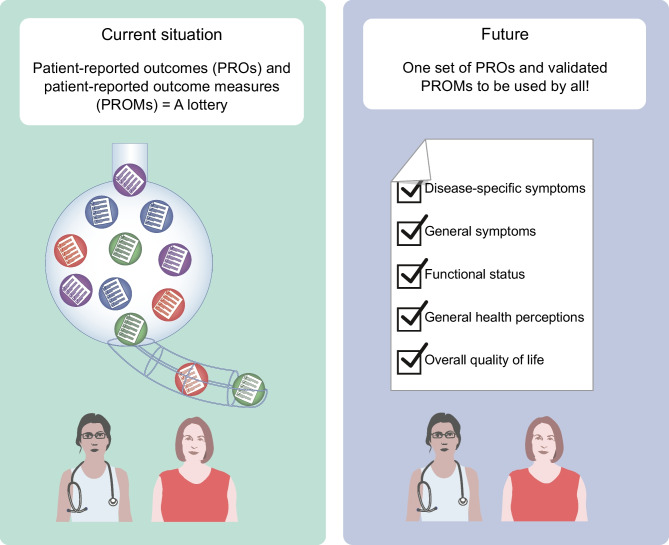

**Supplementary Information:**

The online version contains supplementary material available at 10.1007/s00125-023-05926-3.

## Introduction

In clinical practice, consultations with healthcare providers are often short. In the case of poor emotional well-being, e.g. depressive symptoms, there is limited time available for in-depth discussion. Questionnaires that measure patient-reported outcomes (PROs), so called patient-reported outcome measures (PROMs), can be of help. A PRO was defined by the US Food and Drug Administration (FDA) as ‘any report of the status of a patient’s health condition that comes directly from the patient, without interpretation of the patient’s response by a clinician or anyone else’ [1] (Text box 1). PROMs measuring physical and psychosocial aspects of health and quality of life (QOL) such as physical function or depression, offer complementary information to clinical outcomes such as HbA_1c_, and can be used to inform people with diabetes about the expected course of disease and treatment, for shared decision making, monitoring outcomes and to improve healthcare [2]. Using PROMs does not need to lengthen the consultation time [3].


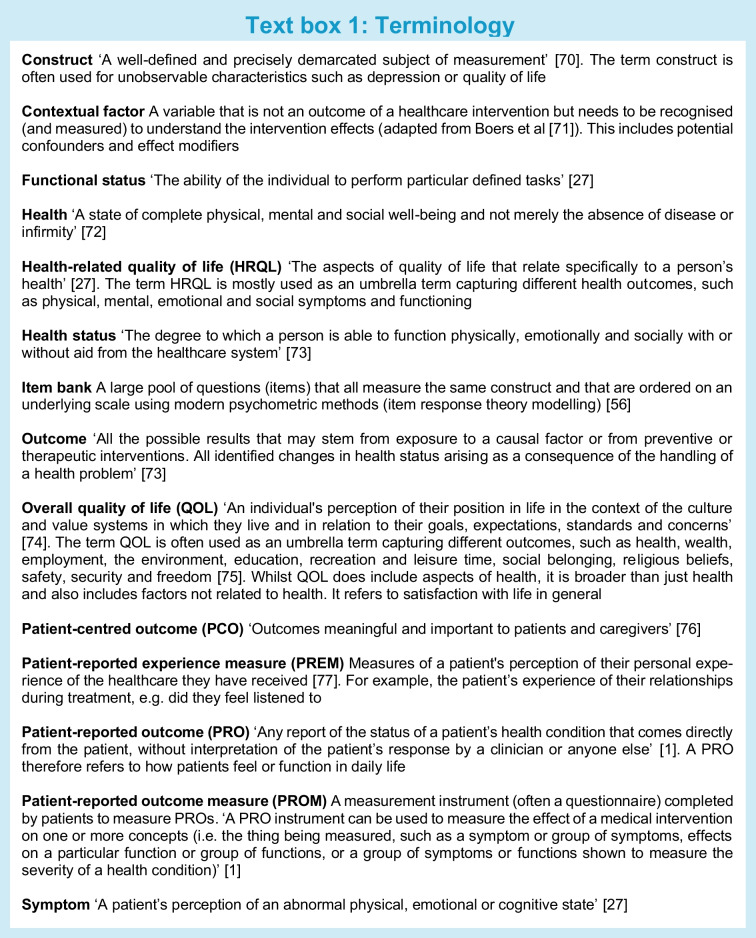


To optimally benefit from using PROMs in research or clinical practice, PROMs should measure those outcomes that are most relevant to people with diabetes. Several initiatives have tried to identify which PROs are most relevant for people with diabetes. An international consortium of people with diabetes, healthcare providers and other relevant stakeholders developed an agreed minimum set of outcomes to be measured in all clinical trials in people with type 2 diabetes (called a core outcome set [COS]). They recommend measuring global QOL and activities of daily living in all clinical trials [4]. The International Consortium for Health Outcomes Measurement (ICHOM) developed a standard set of outcomes to be measured in clinical practice in people with type 1 or type 2 diabetes. They recommend measuring psychological well-being, diabetes distress and depression [5]. Other initiatives recommend yet different PROs [6–9]. Although ‘quality of life’ is often recommended [8], this concept is defined very differently by different people [10]. There are many different questionnaires available that aim to measure QOL or (aspects of) health-related QOL (HRQL); some are generic, some are disease-specific, and they measure many different things, not always restricted to PROs [11]. Furthermore, the validity, reliability and responsiveness to change over time of many of the questionnaires is often unclear or not sufficient [11–26].

The aim of this review is to provide recommendations on the most commonly relevant PROs for adult people with diabetes to measure in clinical practice and research, and good quality PROMs to measure these PROs. We first provide a general conceptual framework of PROs and PROMs. Second, we present a narrative overview of the literature on which PROs are most relevant to measure in people with diabetes. Third, we present an overview of which PROMs have been used in studies involving people with diabetes and what is known about the quality of these PROMs in terms of validity, reliability and responsiveness. In addition, we suggest several well-validated generic PROMs that could be used in people with diabetes. Finally, we provide recommendations and suggestions for the use of PROMs in clinical practice and research.

## A conceptual framework of PROs and PROMs

There is considerable heterogeneity in the definition and operationalisation of the terms ‘QOL’, ‘HRQL’, ‘PRO’ and ‘PROM’ between and within studies [10]. In Text box 1, we provide an overview of commonly used terms and definitions, which we adopted in this paper. We adopt the original definition of a PRO of the FDA [1], which has also been adopted by the European Medicines Agency (EMA). PROs therefore refer to *health* outcomes, including physical, mental, and social symptoms and functioning. Non-health-related constructs, such as overall QOL (which is broader than health), satisfaction, eating behaviour and stigma, are not considered PROs according to the original FDA definition.

**Fig. 1:**
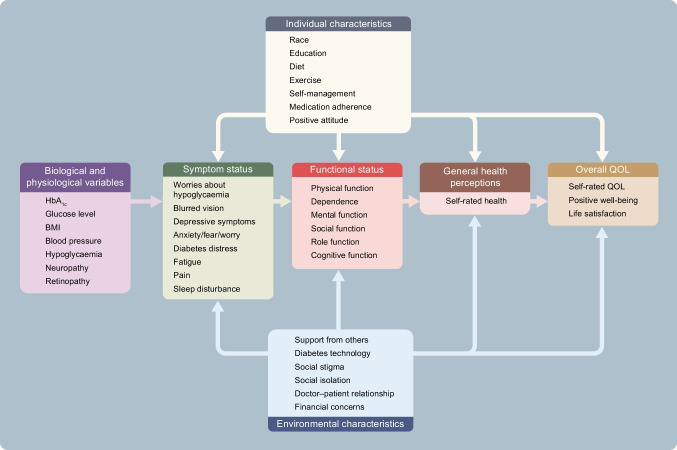
Several examples of relevant health and QOL outcomes for people with diabetes (list is not exhaustive) placed in the model of Wilson and Cleary [27]. This figure is available as a downloadable slide

It is important to conceptualise how different health and QOL outcomes interrelate. Different models have been proposed in the literature. One commonly used model was developed by Wilson and Cleary [27], who distinguish different levels of health and QOL outcomes and relate them to characteristics of the individual and the environment. For illustration, we placed several relevant health and QOL outcomes and characteristics of the individual and the environment for people with diabetes in the Wilson and Cleary model (Fig. 1).

In this model, biological and physiological variables, symptoms, functional status and general health perceptions are considered aspects of health status. Overall QOL is broader than health.

Aspects of health can be thought of as existing on a continuum of increasing biological, social and psychological complexity. Starting from the left-hand side (Fig. 1) are biological and physiological aspects of health such as HbA_1c_, hypoglycaemia, glucose variability or blood pressure. These are measured with clinical measurement instruments, such as glucose sensors, laboratory tests, physical examination, vision tests and imaging techniques.

A biological or physiological abnormality or defect, such as the inability of the pancreas to make insulin, can lead to symptoms, referring to how a patient feels. These could be physical symptoms, such as pain or blurred vision, or emotional or psychological symptoms, such as fear, worry and depressive symptoms. Symptoms are PROs and should be measured with PROMs.

Symptoms can lead to limitations in how an individual functions, in terms of physical function, mental function and social/role function (e.g. performing a job). Functional status can be measured with PROMs, by asking about perceived limitations in functioning, but also with performance-based tests, such as walking tests.

General health perceptions, which refer to the PRO ‘perceived overall health’, are often measured with a single question, e.g. ‘how would you rate your overall health?’, which is a PROM. Finally, overall QOL includes aspects of health, but is broader and also includes factors not related to health, such as material comforts, personal safety and satisfaction with life in general. Overall QOL is actually not a PRO, although (some of) its components can be PROs. Therefore, questionnaires measuring overall QOL are not considered PROMs according to the FDA definition. Wilson and Cleary use the term HRQL as an umbrella term, including symptoms, functional status and general health perceptions [27].

Finally, the model shows that health and QOL outcomes are influenced by contextual factors, i.e. personal factors such as personality, behaviour (diet, medication adherence and physical activity) and coping mechanisms, and environmental factors, such as social support, social stigma and financial aspects.

Commonly used questionnaires in the diabetes field measure different things. Some questionnaires focus on only one level, e.g. symptoms, while others measure outcomes at multiple levels of health, especially questionnaires that aim to measure ‘QOL’ or ‘HRQL’. Some questionnaires classify different outcomes in different subscales, e.g. one subscale for symptoms and another subscale for physical function, but others (undesirably) combine outcomes from different levels into one scale. Many questionnaires include questions or subscales measuring PROs but also contextual factors [11]. These questionnaires are therefore not (entirely) PROMs. Lack of distinction between health and non-health outcomes, between health outcomes and contextual factors, and between PROMs and other questionnaires, results in confusion on what is being measured, lack of content validity of PROMs, difficulty selecting the best PROM for a given study or clinical application, and inability to study causal relationships between health outcomes or the relationship between contextual factors, health outcomes and overall QOL. Text box 2 provides illustrations of measurement issues we encountered in performing systematic reviews of PROMs in people with diabetes [11, 24, 25].


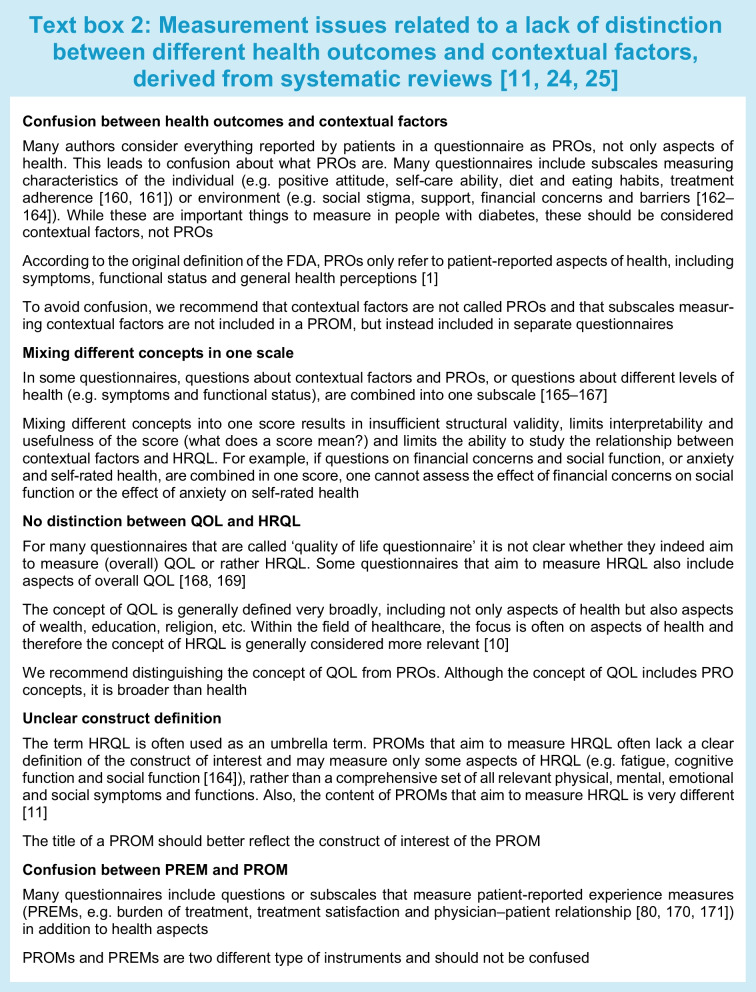


Researchers and clinicians should be aware of the differences between clinical outcomes, PROs, contextual factors and patient experiences, and the fact that all of these concepts are often included in questionnaires or subscales that aim to measure HRQL, QOL or PROs. An illustration is provided in Text box 3. This situation hampers clear interpretation of what is being measured and is a threat to the validity of diabetes research. We cannot, for example, study the influence of self-care behaviour on physical and psychological functioning in people with diabetes if these concepts are measured in one scale and summarised into one score. We cannot appropriately perform or interpret the results of meta-analyses of studies on the effects of certain medication on HRQL, if the HRQL instruments measure all kind of different concepts, some of them not even related to health. All the concepts shown in Fig. 1 can be important to measure, but it is confusing if they are all called PROs or HRQL, and they should not be combined into one scale score.


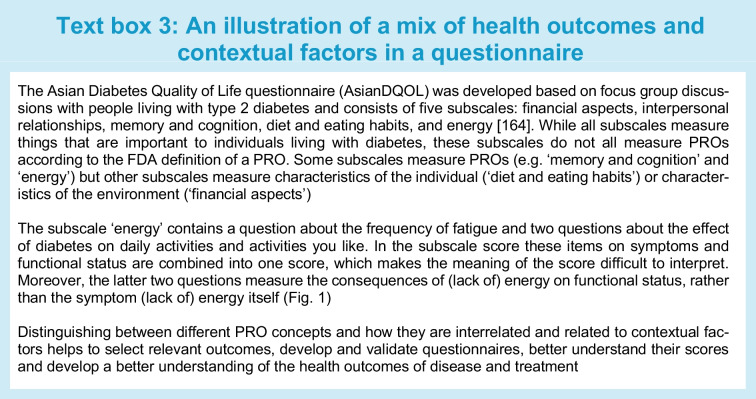


## Most relevant PROs to measure in people with diabetes

It is not clear which PROs are most relevant to measure in diabetes research and clinical practice. Qualitative studies revealed a large number of outcomes considered important by people with diabetes [28–30]. No explicit distinction was made in these studies between PROs, contextual factors and other outcomes, although Dodd et al classified outcomes using the Core Outcome Measures in Effectiveness Trials (COMET) taxonomy [31], where PROs are classified in the category ‘life impact’.

Relevant international guidelines differ in PROs being recommended. Many recommendations state the importance of psychosocial problems. However, a distinction between psychosocial functioning (a PRO) and psychosocial well-being (broader than a PRO) is not made. Harman et al developed a COS to be measured and reported, as a minimum, in all clinical trials in people with type 2 diabetes [4]. A COS often contains PROs but also other relevant (clinical) outcomes. The COS was developed in a Delphi survey with healthcare professionals, people with type 2 diabetes, researchers in the field and healthcare policymakers. Recommended core outcomes to be reported by patients were ‘global QOL’ and ‘activities of daily living’. Global QOL was defined as ‘someone’s overall quality of life, including physical, mental and social well-being’ [4]. This is actually not a PRO because it is broader than health. Activities of daily living was defined as ‘being able to complete usual everyday tasks and activities, including those related to personal care, household tasks or community-based tasks’ [4]. This refers to a PRO.

The ICHOM consortium developed a standard set for people with diabetes types 1 and 2, to be used in clinical practice, also using a consensus approach among experts. It does not state whether people with diabetes were involved. Recommended outcomes are psychological well-being, diabetes distress and depression. Only the latter two are PROs [5]. This recommendation is in line with recommendations from the ADA and the EASD, which state that providers should consider diabetes distress, depression, anxiety, disordered eating (which is not a PRO), cognitive capacities and chronic pain [6, 32–35].

Differences in recommendations are at least partly due to different aims, methodology, and (lack of) involvement of people with diabetes. For example, a COS includes only a minimum set of outcomes to be measured and reported in every clinical trial, while other guidelines might include outcomes that could be relevant to measure in addition in specific trials or in clinical practice.

In summary, there is consensus that the PRO ‘activities of daily living’ (which is conceptually similar to physical function) should be measured in all diabetes trials. There is less consensus on which PROs are additionally relevant to measure in specific trials and which PROs are relevant to measure routinely in clinical practice.

In the meantime, there is increasing evidence from several initiatives that some PROs are relevant for many people, irrespective of their disease (Text box 4) [36–38]. Symptoms such as pain, fatigue or depression are common across diseases. Furthermore, being able to carry out daily activities and social roles is important to most people. The Patient-Reported Outcomes Measurement Information System (PROMIS) domain framework was developed to capture commonly relevant PROs across three broad aspects of physical, mental and social health based on the WHO definition of health [37, 39]. It was developed through literature reviews of well-established instruments, a consensus-building Delphi process among health outcomes experts and statistical analysis. Patients were not involved in the development of the conceptual model, although patient input was captured by reviewing instruments that were developed with patient input [40]. Five subdomains were selected as the initial areas for PROMIS item bank construction: fatigue, pain, emotional distress (later divided into depression, anxiety and anger), physical functioning and social role participation [37].


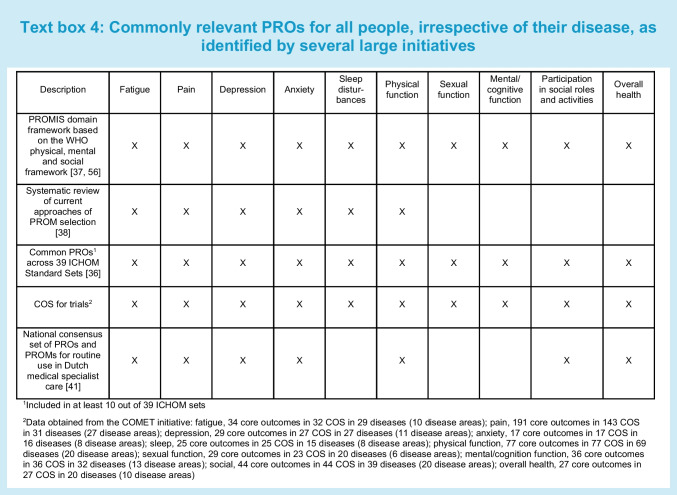


Kroenke et al developed a taxonomy of key pragmatic decisions related to PROM implementation based on literature review, but without patient input [38]. One of the pragmatic issues they address is the selection of generic vs disease-specific PROMs. They noted that some domains are crosscutting in the fact that they occur frequently and often cluster across the majority of medical and mental health disorders, including fatigue, pain, depression, anxiety, sleep and physical function [38].

Terwee et al extracted all PROs and recommended PROMs from 39 ICHOM Standard Sets [36]. Many of these sets were developed with patient input, but not all. More than 300 PROs were categorised into 22 unique PRO concepts. The most commonly included PROs were ability to participate in social roles, physical function, HRQL, pain, depression, general mental health, anxiety and fatigue [36]. The COMET initiative identified similar common PROs included in COS for trials (Text box 4).

In the Netherlands, a national consensus set of PROs and PROMs was recently developed for routine use in Dutch medical specialist care, based on the above mentioned initiative and others, as well as input from patients, healthcare providers and representatives of healthcare organisations. The selected PROs were fatigue, pain, depression, anxiety, physical functioning, social role participation and overall health [41].

Based on these initiatives, we recommend considering the commonly relevant PROs mentioned above to measure in people with diabetes (both type 1 and type 2). These PROs can be supplemented with relevant diabetes-specific symptoms. For example, the WHO report on diabetes lists frequent urination, thirst, feeling hungry (even though you are eating), blurry vision, weight loss (type 1) and tingling hands/feet (type 2) as relevant symptoms of diabetes [42]. In addition, other relevant PROs that are commonly measured could be considered, such as diabetes distress and fear of hypoglycaemia.

## Best PROMs for use in people with diabetes

It is very challenging to identify the best PROMs to measure the above suggested PROs in people with diabetes. At least 16 systematic reviews have been published summarising the available PROMs and their measurement properties for people with diabetes [11–26]. These reviews vary in quality and completeness, while some included selected groups (i.e. only type 1 or type 2 or people with amputations), some focused on only one PRO (e.g. depression), and some were conducted over 10 years ago. As a result, the identified PROMs, evaluation methods, conclusions and recommendations of these reviews vary.

Our systematic review by Langendoen-Gort et al provides the most recent overview of existing PROMs, published up to 31 December 2021, that aim to measure (aspects of) HRQL and that have been validated to at least some extent in people with type 2 diabetes [11]. We identified 116 questionnaires. Not all of these questionnaires actually measure PROs. About half (61) of the 116 questionnaires (also) include items or subscales measuring characteristics of the individual (e.g. aspects of personality and coping) or environment (e.g. social or financial support), or patient experiences and treatment satisfaction. Eight out of the 116 questionnaires measured no PRO at all, even though they claim to measure HRQL [11]. No recommendations were provided on the best PROMs because the measurement properties of the PROMs were not assessed in this review.

The international COnsensus-based Standards for the selection of health Measurement INstruments (COSMIN) initiative developed consensus-based standards and criteria for assessing the quality of PROMs [43, 44]. Nine measurement properties are considered important for PROMs: content validity, structural validity, internal consistency, construct validity, reliability, measurement error, cross-cultural validity, criterion validity (only for comparing different versions of the same PROM) and responsiveness (Table 1) [45]. According to COSMIN, the most important measurement property is content validity [46]. In a second review, we assessed the content validity of 54 of the above mentioned 116 PROMs, containing 150 subscales that were specifically developed for people with type 2 diabetes. Using COSMIN methodology [46], we assessed whether all PROM items measure relevant aspects of the construct the PROM (scale) aims to measure, whether no important aspects are missing and whether the items are interpreted by the person as intended. Most previous reviews did not evaluate content validity, or not in as much detail as the COSMIN methodology recommends. We showed that content validity was rated as sufficient for only 41 out of the 150 (27%) PROM subscales [25]. In Table 1 we provide a narrative summary of the relevant evidence on all measurement properties of these PROM subscales, excluding single items and scales developed for subgroups of people with diabetes (e.g. foot ulcers), classified according to the Wilson and Cleary model [27]. Evidence on the measurement properties other than content validity was extracted from the 16 reviews described above as well as from several main validation papers of the PROMs. We did not find such an evidence synthesis for type 1 diabetes.

**Table 1:** Measurement properties of type 2 diabetes-specific (subscales of) PROMs with sufficient content validity

Name of PROM	Number of items	Content validity [25]^1^	Structural validity	Internal consistency (Cronbach’s alpha)	Construct validity^2^	Test–retest reliability and measurement error	Cross-cultural validity	Responsiveness
Description		Relevance, comprehensiveness and comprehensibility of the items in a PROM	Extent to which the items in a (sub)scale measure only one construct	Interrelatedness among the items of a (sub)scale	Correlations to similar PROMs as expected	Consistency of results when the same PROM is completed at a different point in time	Same construct is being measured across countries	Ability to measure change over time
Diabetes-specific symptoms								
DSSCI [78]	38	⊕⊕	?	0.92	*R*=0.57 with Illness Perception Questionnaire Identity subscale	?	?	?
DSC-R Hypoglycemic symptoms [79]	3	⊕	8-factor model with CFI 0.90 RMSEA 0.05	0.77	HbA_1c_ levels	?	?	Global rating of change
DSC-R Hyperglycemic symptoms [79]	4	⊕	8-factor model with CFI 0.90 RMSEA 0.05	0.79	HbA_1c_ levels	?	?	Global rating of change
DSC-R Cardiovascular symptoms [79]	3	⊕	8-factor model with CFI 0.90 RMSEA 0.05	0.69	BMI	?	?	Global rating of change
DSC-R Neuropathic pain [79]	4	⊕	8-factor model with CFI 0.90 RMSEA 0.05	0.76	*R*=0.38 SF-36 Bodily Pain	?	?	Global rating of change
DSC-R Neuropathic sensoric [79]	6	⊕	8-factor model with CFI 0.90 RMSEA 0.05	0.84	*R*=0.37 SF-36 Bodily Pain	?	?	Global rating of change
DSC-R Ophthalmologic symptoms [79]	5	⊕	8-factor model with CFI 0.90 RMSEA 0.05	0.85	?	?	?	Global rating of change
DQLCTQ/ DQLCTQ-R Frequency of symptoms [80]	7	⊕	?	0.77	HbA_1c_ levels and type of diabetes (T1D vs T2D)	?	?	Metabolic control
DQLCTQ/ DQLCTQ-R Bothersomeness of symptoms [80]	7	⊕	?	0.80	HbA_1c_ levels and type of diabetes (T1D vs T2D)	?	?	Metabolic control
Fatigue								
DQLCTQ/DQLCTQ-R Energy/Fatigue [18, 80]	5	⊕	?	0.85	HbA_1c_ levels and type of diabetes (T1D vs T2D)	0.85	?	Metabolic control
W-BQ12 Energy [81, 82]	4	⊕	Unidimensional model with CFI 0.93	0.77−0.87	*R*=0.85 with SF-36 Vitality	0.80	?	?
Diabetes distress								
DDS Emotional burden [15, 83–86]	5	⊕	?	>0.80	*R*=0.51 with SF-36 Mental Health, *R*=0.48 with SF-36 MCS, *R*=0.52 & 0.44 with HADS Anxiety & Depression, *R*=0.55 with CESD	0.76−0.78 (whole DDS)	?	?
DDS Physician-related distress [15, 83–86]	4	⊕	?	>0.80	?	0.76−0.78 (whole DDS)	?	?
DDS Interpersonal distress [15, 83–86]	3	⊕	?	>0.80	*R*=0.33 with SF-36 Social Functioning, *R*=0.48 with CESD	0.76−0.78 (whole DDS)	?	?
PAID-20 Diabetes-related emotional problems [18, 87–91]	12	⊕	Inconsistent results (different models proposed)	0.93−0.95	*R*=0.53 with HFS, *R*=0.60 with STAI	0.80−0.83	?	Change after interventions
Anxiety								
Diabetes Questionnaire Worries [92, 93]	3	⊕⊕⊕	?	?	*R*=0.41 with SF-36 Mental Health	weighted κ 0.49−0.60 T1D, 0.46−0.52 T2D	?	?
DQLCTQ Worry (HFS) [18, 80]	17	⊕	?	0.94	Self-perceived control	0.73	?	Metabolic control
DQOL Diabetes-related worry [51, 94]	3−7	⊕	?	0.67	*R*=0.46 with PAIS psychological distress, *R*=0.46 with Diabetes-39 Anxiety and Worry scale	0.80	?	?
Physical function								
IWADL [24, 95, 96]	7	⊕⊕	?	0.94	± High	0.91 Measurement error: low	?	Weight loss
Sexual function								
Diabetes-39 Sexual Functioning [97, 98]	3	⊕	⊕	⊕ >0.80, 0.92	?	?	?	?
Emotional function								
DQLCTQ/DQLCT-R Mental health [18, 80]	5	⊕	?	0.82	Self-perceived control	0.83	?	Metabolic control
Social function								
Diabetes Questionnaire Barriers [92, 93]	5	⊕⊕⊕	?	?	*R*=0.42 with SF-36 Social Functioning	Weighted κ 0.41−0.67 T1D; 0.32−0.64 T2D	?	?
Overall self-rated health								
Diabetes Questionnaire How you feel [92, 93]	5	⊕⊕⊕	?	?	*R*=0.56 with SF-36 General Health Perceptions	Weighted κ 0.50−0.65 T1D; 0.40−0.63 T2D	?	?
DQOL Impact [51, 94]	18−27	⊕	?	0.67	*R*=0.50 with Symptom Checklist-90-R	0.89	?	?
SPH Feel Healthy [99]	5	⊕	?	?	?	?	?	?

Data was extracted from 16 systematic reviews [11–26] and some additional validation studies. This is not a comprehensive systematic review, but provides the most relevant evidence on the measurement properties of these PROM scales. No information on criterion validity was found

COSMIN criteria for sufficient measurement properties were used [44]: structural validity consistent factor model with CFI >0.95, RMSEA <0.08; internal consistency (Cronbach’s alpha) >0.70; test–retest reliability (ICC) >0.70; construct validity/responsiveness (correlations with PROMs measuring similar constructs) ≥0.50

^1^ ⊕ Very low, ⊕⊕ low and ⊕⊕⊕ moderate refer to the quality of the evidence (higher quality studies or more available studies lead to higher quality evidence)[46]

^2^ Construct validity: only correlations with PROMs measuring similar constructs are presented

?, no information available; CESD, Center for Epidemiologic Studies Depression scale; CFI, comparative fit index; DDS, Diabetes Distress Screening; DSC-R, Diabetes Symptom Checklist-Revised; DSSCI, Diabetes Symptom Self-Care Inventory; DQLCTQ, Diabetes Quality of Life Clinical Trial Questionnaire; DCLCTQ-R, Diabetes Quality of Life Clinical Trial Questionnaire -Revised; DQOL, Diabetes Quality of Life; HADS, Hospital Anxiety and Depression Scale; HFS, Hypoglycemia Fear Survey; ICC, intraclass correlation coeffient; MCS, Mental Component Summary; PAID, Problem Areas in Diabetes; PAIS, Psychosocial Adjustment of Illness Scale; RMSEA, root mean square error of approximation; SPH, self-perception of health; STAI, State-Trait Anxiety Index; T1D, type 1 diabetes; T2D, type 2 diabetes; W-BQ12, Well-being and Treatment Satisfaction scales

Table 1 shows that none of the existing diabetes-specific PROM scales have been sufficiently validated. COSMIN states that PROMs with evidence for sufficient content validity (any level) and at least low evidence for sufficient structural validity and internal consistency have the potential to be recommended for use [44]. In addition, evidence on reliability (small measurement error) is important, especially for PROMs used in clinical practice. All PROMs measuring disease-specific symptoms showed positive results for internal consistency, but these results cannot be interpreted properly if evidence that the scale is unidimensional is lacking [47]. Also, important information on test–retest reliability and responsiveness is lacking. The Diabetes Symptom Self-Care Inventory (DSSCI) is most promising for measuring diabetes-specific symptoms because it has the best evidence for content validity. For measuring diabetes distress, the Diabetes Distress Scale (DDS) and the Problem Areas in Diabetes (PAID) scale are most promising based on content validity.

For the diabetes-specific PROMs or subscales measuring fatigue, anxiety, physical function, sexual function, emotional function, social function and overall health, evidence on structural validity, test–retest reliability and responsiveness is missing. Therefore, none of these PROMs can be recommended. Considering that these PROs are commonly relevant across medical conditions (Text box 4) and that the content of disease-specific PROMs and generic PROMs measuring the same PRO are often very similar, we recommend using generic PROMs for these PROs.

For these commonly relevant PROs, high-quality generic PROMs exist that are applicable across populations and diseases (Table 2). Not all of these generic PROMs have been validated in people with diabetes (Table 2), but since they showed good measurement properties in other chronic conditions, it may be reasonable to assume that they will also perform well in people with diabetes. We discuss three generic PROMs that are widely used and tested: the 36-Item Short Form Health Survey (SF-36) [48], the WHO Disability Assessment Schedule 2.0 (WHODAS 2.0) [49] and the PROMIS measurement system [39].

**Table 2:** Commonly used, well-validated generic PROMs for measuring common PROs

Fatigue	The 13-item Functional Assessment of Chronic Illness Therapy–Fatigue Scale (FACIT–Fatigue) was originally developed for cancer patients. Most validation studies were therefore performed in cancer patients [100–102], but it has been validated and used across many other conditions. Evidence on structural validity (i.e. whether the scale measures one or two constructs) seems inconsistent. Some evidence for content validity, internal consistency and test–retest reliability was found in Turkish people with diabetes [103]. More information and available language versions can be found on the FACIT website. The PROMIS Fatigue item bank, short forms and CAT have been validated in several general and clinical populations, including people with kidney disease, and appear to be unidimensional [60, 104–107]. Evidence for construct validity, test–retest reliability and responsiveness of the PROMIS Fatigue CAT was found in Dutch people with diabetes (F. Rutters, unpublished results). The PROMIS Fatigue short forms are part of the commonly used PROMIS-29, PROMIS-43, and PROMIS-57 [108], which have been validated across general and clinical populations [108–114]. These measures have been used (but not validated) in people with diabetes in clinical practice and research [65, 115–117]. The FACIT–Fatigue has been adopted by the PROMIS initiative and is now also called the PROMIS SF v1.0 Fatigue 13a. Available language versions of PROMIS can be found on the HealthMeasures website (www.healthmeasures.net/explore-measurement-systems/promis/intro-to-promis/available-translations).
Pain	A single 11-point (i.e. 0–10) numerical rating scale (NRS) for measuring pain intensity was recommended by the Initiative on Methods, Measurement, and Pain Assessment in Clinical Trials (IMMPACT) initiative as a core outcome measure in clinical trials of chronic pain treatments [118]. The NRS has been used (but not validated) in diabetes studies (e.g. Higgins et al [119]). The PROMIS Numeric Rating Scale v1.0–Pain Intensity 1a, for example, is an NRS that can be used as a standalone measure or as part of the commonly used PROMIS Global Health [69], PROMIS-29, PROMIS-43 and PROMIS-57 [108]. The PROMIS Global Health, PROMIS-29 and PROMIS-57 have been used (but not validated) in people with diabetes in clinical practice and research [65, 115–117, 120, 121]. The SF-36 is perhaps the most commonly used generic PROM in the world. It was included in more than 300 systematic reviews of measurement properties of PROMs, included in the COSMIN database [50]. The SF-36 subscale Bodily Pain asks about pain severity and the interference of pain with daily activities. Evidence for internal consistency, construct validity and responsiveness of the SF-36 has been found in people with diabetes (e.g. Huang et al [51], Ahroni and Boyko [52], and Martin et al [122]). Available language versions of the SF-36 can be found in the Patient-Reported Outcome and Quality of Life Instruments Database (PROQOLID) (https://www.qolid.org/instruments/sf_36_sup_r_sup_health_survey_and_sf_36v2_sup_tm_sup_health_survey_sf_36_sup_r_sup_sf_36v2_sup_tm_sup/).
Anxiety	The Generalized Anxiety Disorder-7 (GAD-7) is a brief screening tool developed to identify probable cases of generalised anxiety disorder and assess symptom severity [123]. It has been widely used and validated (e.g. Breedvelt et al [124] and Toussaint et al [125]). Findings regarding its structural validity are mixed, with most studies reporting it to be one scale (including a study in people with diabetes in India [126]), whereas others found two subscales [127]. Available language versions of the GAD-7 can be found on the website of Patient Health Questionnaire (PHQ) Screeners. The Hospital Anxiety and Depression Scale (HADS) was published in 1983 as a self-assessment scale for detecting states of depression and anxiety in a hospital setting [128]. The HADS is widely used and has been extensively validated in many different conditions [50] (some may include people with diabetes, but we found no validation study in only people with diabetes), although evidence on structural validity is inconsistent. The HADS consists of two subscales, measuring anxiety and depression, respectively, although others have suggested that it can be used as one unidimensional scale [129]. More information and available language versions of the HADS can be found on the ePROVIDE website (https://eprovide.mapi-trust.org/instruments/hospital-anxiety-and-depression-scale). The SF-36 subscale Mental health is widely used, and has been validated in people with diabetes (e.g. Huang et al [51], Ahroni and Boyko [52], and Martin et al [122]). The more recently developed PROMIS Anxiety item bank and derivative short forms and CAT were found to be unidimensional and have been validated in several general and clinical populations [130–133]. Evidence for sufficient construct validity, test–retest reliability and responsiveness of the PROMIS Anxiety CAT was found in Dutch people with diabetes (F. Rutters, unpublished results). The PROMIS Anxiety short forms are part of the commonly used PROMIS-29, PROMIS-43, and PROMIS-57 [108] (see above) [65, 115–117].
Depression	The HADS depression subscale is described above. van Dijk et al concluded in a systematic review that the generic Center for Epidemiologic Studies Depression scale (CESD) was best supported for measuring depressive symptoms in people with diabetes [21]. However, evidence on structural validity is inconsistent. Although the CESD is used as a unidimensional scale, most studies found three or four underlying concepts [134]. The CESD was revised to CESD-R in 2004. More information and available language translations can be found on the CESD website (https://cesd-r.com/). The Patient Health Questionnaire (PHQ-9) [135] has been used in more than 5000 studies listed on PubMed. It was included in more than 30 systematic reviews of measurement properties of PROMs [50]. van Dijk found evidence for construct validity, and criterion validity in people with diabetes, but evidence for structural validity was inconsistent [21]. Available language versions of the PHQ-9 can be found on the website of Patient Health Questionnaire (PHQ) Screeners (www.phqscreeners.com/select-screener). The SF-36 subscale Mental health (see above) is widely used, and has been validated in people with diabetes (e.g. Huang et al [51], Ahroni and Boyko [52], and Martin et al [122]). The more recently developed PROMIS Depression item bank and derivative short forms and CAT were found to be unidimensional and have been validated in several general and clinical populations [58, 132, 133, 136–138]. High internal consistency of the PROMIS Depression 8-item short form was found in people with diabetes [139]. Evidence for construct validity, test–retest reliability and responsiveness of the PROMIS Depression CAT was found in Dutch people with diabetes (F. Rutters, unpublished results). The PROMIS Depression short forms are part of the commonly used PROMIS-29, PROMIS-43 and PROMIS-57 [108] (see above), which have been used (but not validated) in people with diabetes in clinical practice and research [65, 115–117].
Sleep disturbances	The Pittsburgh Sleep Quality Index (PSQI) is the most frequently used measure of sleep quality. However, evidence on structural validity was found to be inconsistent [140]. It has been used in more than 5000 studies (PubMed) and was included in 28 systematic reviews of measurement properties of PROMs (https://database.cosmin.nl). More information and available language versions can be found on the ePROVIDE website (https://eprovide.mapi-trust.org/instruments/pittsburgh-sleep-quality-index). The PROMIS Sleep Disturbance and Sleep-Related Impairment item banks and derivative short forms and CAT were found to be unidimensional and have been validated in several general and clinical populations [141–144]. Evidence for construct validity, test–retest reliability and responsiveness of the PROMIS Sleep Disturbance CAT was found in Dutch people with diabetes (F. Rutters, unpublished results). Sufficient responsiveness of the short forms of both PROMIS measures was found in people with type 2 diabetes and sleep apnoea [145]. The PROMIS Sleep Disturbance short forms are part of the commonly used PROMIS-29, PROMIS-43 and PROMIS-57 [108] (see above), which have been used (but not validated) in people with diabetes in clinical practice and research, respectively [65, 115–117].
Physical function	Elsman et al concluded in a systematic review that the Diabetic Foot Ulcer Scale short form (DFS-SF) subscale Dependence/Daily Life (developed for people with diabetes and foot ulcers) and the IWADL could best be used to measure physical functioning in people with type 2 diabetes in research or clinical practice, although both scales have some limitations [24]. More information and available language versions of the DFS and DFS-SF can be found on the ePROVIDE website (https://eprovide.mapi-trust.org/instruments/diabetic-foot-ulcer-scale). The SF-36 subscale Physical Functioning (see above) is probably the most commonly used generic unidimensional physical function subscale and has been validated in people with diabetes (e.g. Huang et al [51], Ahroni and Boyko [52], and Martin et al [122]). The unidimensional PROMIS Physical Function item bank and derivative short forms and CAT are the most commonly used and most often translated measures of the PROMIS system and have been validated in several general and clinical populations, most often in people with musculoskeletal disorders [146–149]. Evidence for construct validity, test–retest reliability and responsiveness of the PROMIS Physical Function CAT was found in Dutch people with diabetes (F. Rutters, unpublished results). The PROMIS Physical Function short forms are part of the commonly used PROMIS-29, PROMIS-43 and PROMIS-57 [108] (see above), which have been used (but not validated) in people with diabetes in clinical practice and research, respectively [65, 115–117].
Sexual function	The most widely used measures of sexual function are the Female Sexual Function Index (FSFI) for women and the International Index of Erectile Function (IIEF) for men. However, conflicting and lack of evidence was found for some of their measurement properties [150, 151]. On the ePROVIDE website more information and available language versions can be found for the FSFI (https://eprovide.mapi-trust.org/instruments/female-sexual-function-index) and IIEF (https://eprovide.mapi-trust.org/instruments/international-index-of-erectile-function). The PROMIS Sexual Function and Satisfaction Profile measures for women and men were developed more recently and have been validated to at least some extent in cancer patients, but not yet in people with diabetes, and they have so far been used less often [152–154].
Cognitive function	The PROMIS Cognitive Function and Cognitive Function–Abilities item banks and derivative short forms and CAT have recently been developed as part of the PROMIS system and have been validated to some extent [155, 156].
Participation in social roles and activities	The SF-36 subscales Physical role functioning and Emotional role functioning are widely used, and have been validated in people with diabetes (e.g. Huang et al [51], Ahroni and Boyko [52], and Martin et al [122]). The WHODAS 2.0 is a generic instrument covering several domains of function and participation. The subscale Participation measure joining in community activities. The WHODAS 2.0 is one of the most widely validated measures of participation [157] and has been used in several large population studies (e.g. Alonso et al [54] and Thorpe et al [55]). It has not been validated in people with diabetes. More information on the WHODAS 2.0 and available language versions can be found on the WHO website (www.who.int/standards/classifications/international-classification-of-functioning-disability-and-health/who-disability-assessment-schedule). The PROMIS Ability to Participate in Social Roles and Activities and PROMIS Satisfaction with Social Roles and Activities item banks and derivative short forms and CAT have been validated in large general population samples and we found them to be unidimensional [158, 159]. Evidence for construct validity, test–retest reliability and responsiveness of the PROMIS Ability to Participate in Social Roles and Activities CAT was found in Dutch people with diabetes (F. Rutters, unpublished results). The PROMIS Ability to Participate in Social Roles and Activities short forms are part of the commonly used PROMIS-29, PROMIS-43 and PROMIS-57 [108] (see above), which have been used (but not validated) in people with diabetes in clinical practice and research, respectively [65, 115–117].
Perceived overall Health	The first item of the SF-36 (see above) refers to perceived overall health. This item was adopted by PROMIS (PROMIS Global01) as part of the PROMIS Global Health [69]. The PROMIS Global Health has been used (but not validated) in people with diabetes [115, 120, 121].

The SF-36, developed in 1992, is the most commonly used generic PROM in the world. It has been extensively validated across medical conditions, illustrated by more than 300 systematic reviews of measurement properties of instruments including this PROM [50]. The SF-36 contains 36 items, divided into eight subscales, measuring physical functioning, bodily pain, role limitations due to physical health problems, role limitations due to personal or emotional problems, emotional well-being, social functioning, energy/fatigue and general health perceptions. Although content validity, structural validity and internal consistency have not been assessed in people with diabetes, evidence for sufficient construct validity and responsiveness has been found in people with diabetes (e.g. Huang et al [51] and Ahroni and Boyko [52]).

The WHODAS 2.0 is a generic instrument covering several domains of function and participation, directly linked to the International Classification of Functioning, Disability and Health (ICF). The original WHO/DAS was published in 1988 and WHODAS 2.0 in 2010 [49]. It includes 36 items, divided into six subscales measuring cognition, mobility, self-care, getting along, life activities and participation [53]. WHODAS 2.0 has been used in large epidemiological studies in people with diabetes [54, 55] but has not been validated in people with diabetes.

The development of PROMIS started in 2004. PROMIS consists of ‘item banks’ instead of fixed PROMs, which has many advantages. An item bank is a large set of items that all measure one PRO (e.g. physical function) and that are ordered on a metric using psychometric methods based on item response theory (IRT) methods [56]. For example, the item ‘are you able to run 5 miles?’ is considered more difficult than the item ‘are you able to get in and out of bed?’ and therefore ordered higher on a physical function metric (if higher scores indicate better function). Individuals get a score on the same metric based on their answers. With items banks, it is not required to administer all items. Instead, a score can be obtained by administering only a subset of items as a short form. The ultimate advantage of item banks is the possibility of computerised adaptive testing (CAT), where after a starting question, the computer selects subsequent questions based on the answers to previous questions. This process continues until a predefined precision, or a maximum number of items is reached. CAT reduces patient burden compared with fixed-item questionnaires [57]. The responsiveness of measures derived from item banks is generally higher than traditional generic PROMs [58–60]. This is important because generic PROMs such as the SF-36 and WHODAS 2.0 generally have limited responsiveness for measuring change over time because the questions are broadly formulated. Item banks and CAT are therefore considered by some to be the future of outcome measurement [56]. Item banks are also sustainable because items can be adapted, removed or added without changing the underlying metric. The PROMIS initiative developed a large variety of item banks for measuring key symptoms (fatigue, pain, sleep disturbance, anxiety and depression), functional status (physical function and the ability to perform social roles and activities) and general health perceptions (global health), which have been translated into more than 60 languages and can be administered as short forms or CAT across a wide range of chronic conditions, enabling efficient and interpretable clinical trial and clinical practice applications of PROs [61]. PROMIS uses a T-score metric, where a mean of 50 represents the average of a reference population (usually a general population). Although content validity, structural validity and internal consistency have not been assessed in people with diabetes, Groeneveld et al were the first to show sufficient construct validity, test–retest reliability and responsiveness of seven PROMIS CATs for measuring physical function, pain interference, fatigue, sleep disturbance, anxiety, depression and ability to participate in social roles and activities in 314 people with type 2 diabetes (F. Rutters, unpublished results).

Finally, there are also high-quality generic PROMs available that measure only one PRO, such as the Functional Assessment of Chronic Illness Therapy (FACIT)–Fatigue Scale, or two PROs, such as the Hospital Anxiety and Depression Scale (HADS) for anxiety and depression, that could be considered. A description of relevant SF-36 and WHODAS subscales, PROMIS measures and some other commonly used generic PROMs that focus on only one or a few PROs and that we consider to have good content validity, is presented in Table 2. A narrative summary of evidence on their measurement properties in general, and any evidence that we could find on the measurement properties in people with diabetes, is presented in Table 2.

We recommend selecting a relevant PROM or a subscale of a PROM from Table 2 for each PRO that one aims to measure in a study or clinical application. The SF-36 and WHODAS 2.0 do not need to be administered in total and scales from different PROMs can be mixed based on preferences for a specific context of use. The PROMIS measures are attractive because they take advantage of the modern psychometric technique of IRT, which makes them precise, patient-friendly and short, and they allow for comparisons between disease groups, including those without diabetes and another chronic condition. Another advantage is that these scales are unidimensional, in contrast to several of the other measures mentioned in Table 2. Unidimensional scales measure only one construct, and scores are therefore easier and more valid to interpret.

The generic PROM scales do not assess diabetes-specific constructs such as diabetes distress, and for many studies it can be important to add disease-specific PROMs that measure diabetes-specific symptoms and other relevant diabetes-specific PROs, such as diabetes distress. A combination of disease-specific PROMs for measuring disease-specific symptoms and generic PROMs for measuring general symptoms, functioning and perceived overall health, seems most useful.

## Future: where should we be going?

There is a need for further standardisation of PROs and PROMs in the field of diabetes. We recommend researchers and clinicians consider measuring disease-specific symptoms, general symptoms, functional status and general health perceptions. We recommend further validation of diabetes-specific PROMs that have sufficient content validity for measuring diabetes-specific symptoms and diabetes distress. In addition, we recommend using generic PROMs for measuring commonly relevant PROs. In particular, the use of item banks and CAT, such as those of the PROMIS system, offer many potential benefits for measuring commonly relevant PROs. The main advantages are efficient measurement with minimal number of items yet providing reliable scores; flexible measurement because items can be used interchangeably; and precise measurement due to low measurement error. It is also possible to convert scores of many traditional PROMs to the corresponding PROMIS metric (see for example Bingham et al [62]). PROMIS is rapidly being adopted and used across diseases and countries [63]. Koh et al confirmed that PROMIS might provide a generic solution to measure PROs in the field of diabetes. PROMIS covered five of six themes, 15 of 30 subthemes and 19 of 35 codes that were identified by people living with diabetes as important [28].

PROMs are not yet routinely used in the field of diabetes. A systematic review showed a sparse use of PROMs to assess depressive symptoms and distress during routine clinical care in adults with type 2 diabetes [64]. Scholle et al [65] were the first to study the effect of implementing the PROMIS-29 in routine care for people with diabetes. They reported some challenges understanding the PROMIS scales, but also saw the PROM process as an opportunity to increase their engagement in the treatment and management of their diabetes [65]. Preliminary qualitative data from our group showed that Dutch people living with type 2 diabetes found PROMIS CATs acceptable and indicated they could be an efficient way to start the conversation with a healthcare provider as well as provide people with diabetes with more confidence (F. Rutters, unpublished results). However, participants all felt that ‘questionnaires should never replace personal consultations with the physician’ (F. Rutters, unpublished results). To support healthcare providers with the selection and implementation of PROs and PROMs in clinical practice, several practical guidelines exist (e.g. van der Wees et al [66] and Aaronson et al [67]).

The COS developed for clinical trials in people with type 2 diabetes recommended core outcomes but not yet core outcome measurement instruments. This paper suggests the Impact of Weight on Activities of Daily Living questionnaire (IWADL), SF-36 subscale physical functioning and particularly PROMIS Physical Function measures for measuring the core outcome ‘activities of daily living’. The core outcome ‘global QOL’ is not considered a PRO, but nevertheless relevant to measure, for example, with the WHO well-being index (WHO-5, a short self-reported measure of current mental well-being [68]) or the PROMIS Global02 item (a single item addressing overall QOL, included in the PROMIS Global Health [69]). However, consensus among people with diabetes and healthcare providers is needed before making a final recommendation.

A limitation of this study is that no people with diabetes were involved. Our recommendations are based on literature and our own experiences as researchers with different backgrounds and clinicians. Second, this is not a systematic review of all disease-specific and generic PROs and PROMs that could be used in people with diabetes, and their measurement properties. Additionally, our review focusses predominantly on PROMs for adults with diabetes. However, we hope this paper provides sufficient evidence and recommendations to improve the current state of PROs and PROMs use in the field of diabetes, to improve healthcare and ultimately, improve the QOL of people living with diabetes.

## Supplementary Information

Below is the link to the electronic supplementary material.

Supplementary file1 (PPTX 276 KB)

## Abbreviations

CAT: Computerised adaptive testing
COMET: Core Outcome Measures in Effectiveness Trials
COS: Core outcome set
COSMIN: COnsensus-based Standards for the selection of health Measurement INstruments
FACIT–Fatigue: Functional Assessment of Chronic Illness Therapy–Fatigue Scale
FDA: Food and Drug Administration
HRQL: Health-related quality of life
ICHOM: International Consortium of Health Outcomes Measurement
IRT: Item response theory
IWADL: Impact of Weight on Activities of Daily Living Questionnaire
PROs: Patient-reported outcomes
PROMIS: Patient-Reported Outcomes Measurement Information System
PROMs: Patient-reported outcome measures
QOL: Quality of life
SF-36: 36-Item Short Form Health Survey
WHODAS: WHO Disability Assessment Schedule

## References

[1] U.S. Department of Health and Human Services, Food and Drug Administration, Center for Drug Evaluation and Research (CDER), Center for Biologics Evaluation and Research (CBER), Center for Devices and Radiological Health (CDRH) (2009) Guidance for Industry. Patient-Reported Outcome Measures: Use in Medical Product Development to Support Labeling Claims. https://www.fda.gov/regulatory-information/search-fda-guidance-documents/patient-reported-outcome-measures-use-medical-product-development-support-labeling-claims

[2] Greenhalgh J (2009). The applications of PROs in clinical practice: what are they, do they work, and why?. Qual Life Res.

[3] Engelen V, Detmar S, Koopman H (2012). Reporting health-related quality of life scores to physicians during routine follow-up visits of pediatric oncology patients: is it effective?. Pediatr Blood Cancer.

[4] Harman NL, Wilding JPH, Curry D (2019). Selecting core outcomes for randomised effectiveness trials in type 2 diabetes (SCORE-IT): a patient and healthcare professional consensus on a core outcome set for type 2 diabetes. BMJ Open Diabetes Res Care.

[5] ICHOM (2019) Type 1 and Type 2 Diabetes in Adults. DATA COLLECTION REFERENCE GUIDE. Available from https://connect.ichom.org/patient-centered-outcome-measures/diabetes/ Accessed 30 Mar 2023

[6] Young-Hyman D, de Groot M, Hill-Briggs F, Gonzalez JS, Hood K, Peyrot M (2016). Psychosocial care for people with diabetes: a position statement of the american diabetes association. Diabetes Care.

[7] Skovlund SE, Troelsen LH, Klim L, Jakobsen PE, Ejskjaer N (2021). The participatory development of a national core set of person-centred diabetes outcome constructs for use in routine diabetes care across healthcare sectors. Res Involv Engagem.

[8] Dodd S, Harman N, Taske N, Minchin M, Tan T, Williamson PR (2020). Core outcome sets through the healthcare ecosystem: the case of type 2 diabetes mellitus. Trials.

[9] Byrne M, O'Connell A, Egan AM (2017). A core outcomes set for clinical trials of interventions for young adults with type 1 diabetes: an international, multi-perspective Delphi consensus study. Trials.

[10] Costa DSJ, Mercieca-Bebber R, Rutherford C, Tait MA, King MT (2021). How is quality of life defined and assessed in published research?. Qual Life Res.

[11] Langendoen-Gort M, Groeneveld L, Prinsen CAC (2022). Patient-reported outcome measures for assessing health-related quality of life in people with type 2 diabetes: A systematic review. Rev Endocr Metab Disord.

[12] Chen YT, Tan YZ, Cheen M, Wee HL (2019). Patient-reported outcome measures in registry-based studies of type 2 diabetes mellitus: a systematic review. Curr Diabetes Rep.

[13] El Achhab Y, Nejjari C, Chikri M, Lyoussi B (2008). Disease-specific health-related quality of life instruments among adults diabetic: a systematic review. Diabetes Res Clin Pract.

[14] Garratt AM, Schmidt L, Fitzpatrick R (2002). Patient-assessed health outcome measures for diabetes: a structured review. Diabetic Med J Br Diabetic Assoc.

[15] Lee J, Lee EH, Kim CJ, Moon SH (2015). Diabetes-related emotional distress instruments: a systematic review of measurement properties. Int J Nurs Stud.

[16] Luscombe FA (2000). Health-related quality of life measurement in type 2 diabetes. Value Health.

[17] Martin-Delgado J, Guilabert M, Mira-Solves J (2021). Patient-reported experience and outcome measures in people living with diabetes: a scoping review of instruments. Patient.

[18] Oluchi SE, Manaf RA, Ismail S, Kadir Shahar H, Mahmud A, Udeani TK (2021). Health related quality of life measurements for diabetes: a systematic review. Int J Environ Res Public Health.

[19] Palamenghi L, Carlucci MM, Graffigna G (2020). Measuring the quality of life in diabetic patients: a scoping review. J Diabetes Res.

[20] Roborel de Climens A, Tunceli K, Arnould B (2015). Review of patient-reported outcome instruments measuring health-related quality of life and satisfaction in patients with type 2 diabetes treated with oral therapy. Curr Med Res Opin.

[21] van Dijk SEM, Adriaanse MC, van der Zwaan L (2018). Measurement properties of depression questionnaires in patients with diabetes: a systematic review. Qual Life Res.

[22] Vieta A, Badia X, Sacristan JA (2011). A systematic review of patient-reported and economic outcomes: value to stakeholders in the decision-making process in patients with type 2 diabetes mellitus. Clin Ther.

[23] Wee PJL, Kwan YH, Loh DHF (2021). Measurement properties of patient-reported outcome measures for diabetes: systematic review. J Med Int Res.

[24] Elsman EBM, Mokkink LB, Langendoen-Gort M (2022). Systematic review on the measurement properties of diabetes-specific patient-reported outcome measures (PROMs) for measuring physical functioning in people with type 2 diabetes. BMJ Open Diabetes Res Care.

[25] Terwee CB, Elders PJM, Langendoen-Gort M (2022). Content Validity of Patient-Reported Outcome Measures Developed for Assessing Health-Related Quality of Life in People with Type 2 Diabetes Mellitus: a Systematic Review. Current diabetes reports.

[26] Carlton J, Leaviss J, Pouwer F (2021). The suitability of patient-reported outcome measures used to assess the impact of hypoglycaemia on quality of life in people with diabetes: a systematic review using COSMIN methods. Diabetologia.

[27] Wilson IB, Cleary PD (1995). Linking clinical variables with health-related quality of life. A conceptual model of patient outcomes. JAMA.

[28] Koh O, Lee J, Tan ML (2014). Establishing the thematic framework for a diabetes-specific health-related quality of life item bank for use in an english-speaking asian population. PLoS One.

[29] Svedbo Engstrom M, Leksell J, Johansson UB, Gudbjornsdottir S (2016). What is important for you? A qualitative interview study of living with diabetes and experiences of diabetes care to establish a basis for a tailored Patient-Reported Outcome Measure for the Swedish National Diabetes Register. BMJ Open.

[30] Gorst SL, Young B, Williamson PR, Wilding JPH, Harman NL (2019). Incorporating patients' perspectives into the initial stages of core outcome set development: a rapid review of qualitative studies of type 2 diabetes. BMJ Open Diabetes Res Care.

[31] Dodd S, Clarke M, Becker L, Mavergames C, Fish R, Williamson PR (2018). A taxonomy has been developed for outcomes in medical research to help improve knowledge discovery. J Clin Epidemiol.

[32] Draznin B, Aroda VR, Bakris G (2022). 5. Facilitating Behavior Change and Well-being to Improve Health Outcomes: Standards of Medical Care in Diabetes-2022. Diabetes Care.

[33] Holt RIG, DeVries JH, Hess-Fischl A (2021). The management of type 1 diabetes in adults. A consensus report by the American Diabetes Association (ADA) and the European Association for the Study of Diabetes (EASD). Diabetes Care.

[34] Speight J, Hendrieckx C, Pouwer F, Skinner TC, Snoek FJ (2020). Back to the future: 25 years of 'Guidelines for encouraging psychological well-being' among people affected by diabetes. Diabet Med.

[35] International Diabetes Federation (2017) Recommendations for managing Type 2 Diabetes in Primary Care. Available from www.idf.org/managing-type2-diabetes. Accessed 30 Mar 2023

[36] Terwee CB, Zuidgeest M, Vonkeman HE, Cella D, Haverman L, Roorda LD (2021). Common patient-reported outcomes across ICHOM Standard Sets: the potential contribution of PROMIS®. BMC Med Inform Decis Mak.

[37] Cella D, Yount S, Rothrock N (2007). The Patient-Reported Outcomes Measurement Information System (PROMIS): progress of an NIH Roadmap cooperative group during its first two years. Med Care.

[38] Kroenke K, Miksch TA, Spaulding AC (2022). Choosing and using patient-reported outcome measures in clinical practice. Arch Phys Med Rehabil.

[39] Cella D, Riley W, Stone A (2010). The Patient-Reported Outcomes Measurement Information System (PROMIS) developed and tested its first wave of adult self-reported health outcome item banks: 2005–2008. J Clin Epidemiol.

[40] DeWalt DA, Rothrock N, Yount S, Stone AA (2007). Evaluation of item candidates: the PROMIS qualitative item review. Med Care.

[41] Oude Voshaar MA, Terwee CB, Haverman L (2023). Development of a standardized set of generic set PROs and PROMs for Dutch medical specialist care. A consensus based co-creation approach. Qual Life Res.

[42] World Health Organization (2016) Global report on diabetes. Available from https://www.who.int/publications/i/item/9789241565257 Accessed 30 Mar 2023

[43] Mokkink LB, Terwee CB, Patrick DL (2010). The COSMIN checklist for assessing the methodological quality of studies on measurement properties of health status measurement instruments: an international Delphi study. Qual Life Res.

[44] Prinsen CAC, Mokkink LB, Bouter LM (2018). COSMIN guideline for systematic reviews of patient-reported outcome measures. Qual Life Res.

[45] Mokkink LB, Terwee CB, Patrick DL (2010). The COSMIN study reached international consensus on taxonomy, terminology, and definitions of measurement properties for health-related patient-reported outcomes. J Clin Epidemiol.

[46] Terwee CB, Prinsen CAC, Chiarotto A (2018). COSMIN methodology for evaluating the content validity of patient-reported outcome measures: a Delphi study. Qual Life Res.

[47] Cortina JM (1993). What is coefficient alpha? An examination of theory and applications. J Appl Psychol.

[48] Ware JE, Sherbourne CD (1992). The MOS 36-item short-form health survey (SF-36). I. Conceptual framework and item selection. Med Care.

[49] Ustun TB, Kostanjesek N, Chatterji S, Rehm J, World Health Organization (2010) Measuring health and disability: manual for WHO Disability Assessment Schedule (WHODAS 2.0). World Health Organization, Geneva

[50] COSMIN database of systematic reviews of outcome measurement instruments. Available from http://database.cosmin.nl/. Accessed 30 Mar 2023

[51] Huang IC, Hwang CC, Wu MY, Lin W, Leite W, Wu AW (2008). Diabetes-specific or generic measures for health-related quality of life? Evidence from psychometric validation of the D-39 and SF-36. Value Health.

[52] Ahroni JH, Boyko EJ (2000). Responsiveness of the SF-36 among veterans with diabetes mellitus. J Diabetes Complications.

[53] World Health Organization. WHO Disability Assessment Schedule 2.0 (WHODAS 2.0). https://www.who.int/standards/classifications/international-classification-of-functioning-disability-and-health/who-disability-assessment-schedule. Accessed 30 Mar 2023

[54] Alonso J, Angermeyer MC, Bernert S (2004). Disability and quality of life impact of mental disorders in Europe: results from the European Study of the Epidemiology of Mental Disorders (ESEMeD) project. Acta Psychiatr Scand Suppl.

[55] Thorpe LE, Greene C, Freeman A (2015). Rationale, design and respondent characteristics of the 2013–2014 New York City Health and Nutrition Examination Survey (NYC HANES 2013–2014). Prev Med Rep.

[56] Cella D, Gershon R, Lai JS, Choi S (2007). The future of outcomes measurement: item banking, tailored short-forms, and computerized adaptive assessment. Qual Life Res.

[57] Chakravarty EF, Bjorner JB, Fries JF (2007). Improving patient reported outcomes using item response theory and computerized adaptive testing. J Rheumatol.

[58] Flens G, Terwee CB, Smits N (2022). Construct validity, responsiveness, and utility of change indicators of the Dutch-Flemish PROMIS item banks for depression and anxiety administered as computerized adaptive test (CAT): A comparison with the Brief Symptom Inventory (BSI). Psychol Assess.

[59] Hung M, Saltzman CL, Greene T (2018). Evaluating instrument responsiveness in joint function: The HOOS JR, the KOOS JR, and the PROMIS PF CAT. J Orthop Res.

[60] Kamudoni P, Johns J, Cook KF (2022). A comparison of the measurement properties of the PROMIS Fatigue (MS) 8a against legacy fatigue questionnaires. Mult Scler Relat Disord.

[61] Cella D, Hays RD (2022). A patient reported outcome ontology: conceptual issues and challenges addressed by the patient-reported outcomes measurement information system(®) (PROMIS(®)). Patient Relat Outcome Meas.

[62] Bingham CO, Bartlett SJ, Kannowski C, Sun L, DeLozier AM, Cella D (2021). Conversion of functional assessment of chronic illness therapy-fatigue to patient-reported outcomes measurement information system fatigue scores in two phase III baricitinib rheumatoid arthritis trials. Arthritis Care Res (Hoboken).

[63] Smith AW, Jensen RE (2019). Beyond methods to applied research: realizing the vision of PROMIS®. Health Psychol.

[64] McMorrow R, Hunter B, Hendrieckx C (2022). Effect of routinely assessing and addressing depression and diabetes distress on clinical outcomes among adults with type 2 diabetes: a systematic review. BMJ Open.

[65] Scholle SH, Morton S, Homco J (2018). Implementation of the PROMIS-29 in routine care for people with diabetes: challenges and opportunities. J Ambul Care Manag.

[66] van der Wees PJ, Verkerk EW, Verbiest MEA (2019). Development of a framework with tools to support the selection and implementation of patient-reported outcome measures. J Patient Rep Outcomes.

[67] Aaronson N, Elliott T, Greenhalgh J et al (2015) User’s guide to implementing patient-reported outcomes assessment in clinical practice. International Society for Quality of Life Research, Milwaukee, WI 10.1007/s11136-011-0054-x 22048932

[68] Topp CW, Østergaard SD, Søndergaard S, Bech P (2015). The WHO-5 Well-Being Index: a systematic review of the literature. Psychother Psychosom.

[69] Hays RD, Bjorner JB, Revicki DA, Spritzer KL, Cella D (2009). Development of physical and mental health summary scores from the patient-reported outcomes measurement information system (PROMIS) global items. Qual Life Res.

[70] de Vet HCW, Terwee CB, Mokkink LB, Knol DL (2011). Measurement in medicine.

[71] Boers M, Kirwan JR, Wells G (2014). Developing core outcome measurement sets for clinical trials: OMERACT filter 2.0. J Clin Epidemiol.

[72] World Health Organization (1948) Available from https://www.who.int/about/governance/constitution Accessed 30 Mar 2023

[73] Porta M (2014). A dictionary of epidemiology.

[74] Mayo NE (2015) Dictionary of quality of life and health outcomes measurement. International Society for Quality of Life Research (ISOQOL), Milwaukee, WI

[75] Nussbaum M, Sen A (1993). The quality of life.

[76] Frank L, Basch E, Selby JV (2014). The PCORI perspective on patient-centered outcomes research. Jama.

[77] Jamieson Gilmore K, Corazza I, Coletta L, Allin S (2022). The uses of patient reported experience measures in health systems: a systematic narrative review. Health Policy.

[78] Garcia AA (2011). The diabetes symptom self-care inventory: development and psychometric testing with Mexican Americans. J Pain Symptom Manage.

[79] Arbuckle RA, Humphrey L, Vardeva K (2009). Psychometric evaluation of the Diabetes Symptom Checklist-Revised (DSC-R)–a measure of symptom distress. Value Health.

[80] Shen W, Kotsanos JG, Huster WJ, Mathias SD, Andrejasich CM, Patrick DL (1999). Development and validation of the Diabetes Quality of Life Clinical Trial Questionnaire. Med Care.

[81] Pouwer F, Snoek FJ, van der Ploeg HM, Ader HJ, Heine RJ (2000). The well-being questionnaire: evidence for a three-factor structure with 12 items (W-BQ12). Psychol Med.

[82] Pouwer F, van der Ploeg HM, Ader HJ, Heine RJ, Snoek FJ (1999). The 12-item well-being questionnaire. An evaluation of its validity and reliability in Dutch people with diabetes. Diabetes Care.

[83] Joensen LE, Tapager I, Willaing I (2013). Diabetes distress in Type 1 diabetes–a new measurement fit for purpose. Diabetic Med J Br Diabetic Assoc.

[84] Graue M, Haugstvedt A, Wentzel-Larsen T, Iversen MM, Karlsen B, Rokne B (2012). Diabetes-related emotional distress in adults: reliability and validity of the Norwegian versions of the Problem Areas in Diabetes Scale (PAID) and the Diabetes Distress Scale (DDS). Int J Nurs Stud.

[85] Polonsky WH, Fisher L, Earles J (2005). Assessing psychosocial distress in diabetes: development of the diabetes distress scale. Diabetes Care.

[86] Batais MA, Alosaimi FD, AlYahya AA (2021). Translation, cultural adaptation, and evaluation of the psychometric properties of an Arabic diabetes distress scale: A cross sectional study from Saudi Arabia. Saudi Med J.

[87] Welch G, Weinger K, Anderson B, Polonsky WH (2003). Responsiveness of the Problem Areas In Diabetes (PAID) questionnaire. Diabet Med.

[88] Welch GW, Jacobson AM, Polonsky WH (1997). The problem areas in diabetes scale. An evaluation of its clinical utility. Diabetes Care.

[89] Snoek FJ, Pouwer F, Welch GW, Polonsky WH (2000). Diabetes-related emotional distress in Dutch and U.S. diabetic patients: cross-cultural validity of the problem areas in diabetes scale. Diabetes Care.

[90] Schmitt A, Reimer A, Kulzer B, Haak T, Ehrmann D, Hermanns N (2016). How to assess diabetes distress: comparison of the Problem Areas in Diabetes Scale (PAID) and the Diabetes Distress Scale (DDS). Diabetic Med J Br Diabetic Assoc.

[91] Siaw MY, Tai BB, Lee JY (2017). Psychometric properties of the Chinese version of the Problem Areas in Diabetes scale (SG-PAID-C) among high-risk polypharmacy patients with uncontrolled type 2 diabetes in Singapore. J Diabetes Investig.

[92] Svedbo Engstrom M, Leksell J, Johansson UB (2018). A disease-specific questionnaire for measuring patient-reported outcomes and experiences in the Swedish National Diabetes Register: Development and evaluation of content validity, face validity, and test-retest reliability. Patient Educ Couns.

[93] Svedbo Engström M, Leksell J, Johansson UB (2020). New Diabetes Questionnaire to add patients' perspectives to diabetes care for adults with type 1 and type 2 diabetes: nationwide cross-sectional study of construct validity assessing associations with generic health-related quality of life and clinical variables. BMJ Open.

[94] Jacobson (1988). Reliability and validity of a diabetes quality-of-life measure for the diabetes control and complications trial (DCCT). The DCCT Research Group. Diabetes Care.

[95] Hayes RP, Nelson DR, Meldahl ML, Curtis BH (2011). Ability to perform daily physical activities in individuals with type 2 diabetes and moderate obesity: a preliminary validation of the Impact of Weight on Activities of Daily Living Questionnaire. Diabetes Technol Ther.

[96] Hayes RP, Schultz EM, Naegeli AN, Curtis BH (2012). Test-retest, responsiveness, and minimal important change of the ability to perform physical activities of daily living questionnaire in individuals with type 2 diabetes and obesity. Diabetes Technol Ther.

[97] Boyer JG, Earp JA (1997). The development of an instrument for assessing the quality of life of people with diabetes. Diabetes-39. Med Care.

[98] Khader YS, Bataineh S, Batayha W (2008). The Arabic version of Diabetes-39: psychometric properties and validation. Chronic Illn.

[99] Rao PR, Shobhana R, Lavanya A, Padma C, Vijay V, Ramachandran A (2005). Development of a reliable and valid psychosocial measure of self-perception of health in type 2 diabetes. J Assoc Phys India.

[100] Machado MO, Kang NC, Tai F (2021). Measuring fatigue: a meta-review. Int J Dermatol.

[101] King MT, Agar M, Currow DC, Hardy J, Fazekas B, McCaffrey N (2020). Assessing quality of life in palliative care settings: head-to-head comparison of four patient-reported outcome measures (EORTC QLQ-C15-PAL, FACT-Pal, FACT-Pal-14, FACT-G7). Support Care Cancer.

[102] Luckett T, King M, Butow P, Friedlander M, Paris T (2010). Assessing health-related quality of life in gynecologic oncology: a systematic review of questionnaires and their ability to detect clinically important differences and change. Int J Gynecol Cancer.

[103] Çinar D, Yava A (2018). Validity and reliability of functional assessment of chronic illness treatment-fatigue scale in Turkish patients with type 2 diabetes. Endocrinol Diabetes Nutr (Engl Ed).

[104] Cella D, Lai JS, Jensen SE (2016). PROMIS fatigue item bank had clinical validity across diverse chronic conditions. J Clin Epidemiol.

[105] Lai J-S, Cella D, Choi S (2011). How item banks and their application can influence measurement practice in rehabilitation medicine: a PROMIS fatigue item bank example. Arch Phys Med Rehabil.

[106] Terwee CB, Elsman EBM, Roorda LD (2022). Towards standardization of fatigue measurement: psychometric properties and reference values of the PROMIS Fatigue item bank in the Dutch general population. Res Methods Med Health Sciences.

[107] van der Willik EM, van Breda F, van Jaarsveld BC (2022). Validity and reliability of Patient-Reported Outcomes Measurement Information System (PROMIS®) using Computerized Adaptive Testing (CAT) in patients with advanced chronic kidney disease. Nephrol Dial Transplant.

[108] Cella D, Choi SW, Condon DM (2019). PROMIS(®) adult health profiles: efficient short-form measures of seven health domains. Value Health.

[109] Elsman EBM, Roorda LD, Smidt N, de Vet HCW, Terwee CB (2022). Measurement properties of the Dutch PROMIS-29 v2.1 profile in people with and without chronic conditions. Qual Life Res.

[110] Rose AJ, Bayliss E, Huang W (2018). Evaluating the PROMIS-29 v2.0 for use among older adults with multiple chronic conditions. Qual Life Res.

[111] Coste J, Rouquette A, Valderas JM, Rose M, Leplège A (2018). The French PROMIS-29. Psychometric validation and population reference values. Rev Epidemiol Sante Publique.

[112] Kang D, Lim J, Kim BG (2021). Psychometric validation of the Korean Patient-Reported Outcome Measurement Information System (PROMIS)-29 Profile V2.1 among patients with chronic pulmonary diseases. J Thorac Dis.

[113] Cai T, Wu F, Huang Q (2022). Validity and reliability of the Chinese version of the Patient-Reported Outcomes Measurement Information System adult profile-57 (PROMIS-57). Health Qual Life Outcomes.

[114] Rimehaug SA, Kaat AJ, Nordvik JE, Klokkerud M, Robinson HS (2022). Psychometric properties of the PROMIS-57 questionnaire, Norwegian version. Qual Life Res.

[115] Jiwani R, Wang J, Berndt A (2020). Changes in patient-reported outcome measures with a technology-supported behavioral lifestyle intervention among patients with type 2 diabetes: pilot randomized controlled clinical trial. JMIR Diabetes.

[116] Homco J, Rodriguez K, Bardach DR (2019). Variation and change over time in PROMIS-29 survey results among primary care patients with type 2 diabetes. J Patient Cent Res Rev.

[117] Ee C, de Courten B, Avard N (2020). Shared medical appointments and mindfulness for type 2 diabetes-a mixed-methods feasibility study. Front Endocrinol (Lausanne).

[118] Dworkin RH, Turk DC, Farrar JT (2005). Core outcome measures for chronic pain clinical trials: IMMPACT recommendations. Pain.

[119] Higgins DM, Heapy AA, Buta E (2022). A randomized controlled trial of cognitive behavioral therapy compared with diabetes education for diabetic peripheral neuropathic pain. J Health Psychol.

[120] Martin M, Patterson J, Allison M, O'Connor BB, Patel D (2021). The influence of baseline hemoglobin a1c on digital health coaching outcomes in adults with type 2 diabetes: real-world retrospective cohort study. JMIR Diabetes.

[121] Patil SJ, Tallon E, Wang Y (2022). Effect of Stanford youth diabetes coaches' program on youth and adults in diverse communities. Fam Commun Health.

[122] Martin ML, Patrick DL, Gandra SR (2011). Content validation of two SF-36 subscales for use in type 2 diabetes and non-dialysis chronic kidney disease-related anemia. Qual Life Res.

[123] Spitzer RL, Kroenke K, Williams JB, Löwe B (2006). A brief measure for assessing generalized anxiety disorder: the GAD-7. Arch Intern Med.

[124] Breedvelt JJF, Zamperoni V, South E (2020). A systematic review of mental health measurement scales for evaluating the effects of mental health prevention interventions. Eur J Public Health.

[125] Toussaint A, Hüsing P, Gumz A (2020). Sensitivity to change and minimal clinically important difference of the 7-item Generalized Anxiety Disorder Questionnaire (GAD-7). J Affect Disord.

[126] De Man J, Absetz P, Sathish T (2021). Are the PHQ-9 and GAD-7 Suitable for Use in India? A Psychometric Analysis. Front Psychol.

[127] Moreno E, Muñoz-Navarro R, Medrano LA (2019). Factorial invariance of a computerized version of the GAD-7 across various demographic groups and over time in primary care patients. J Affect Disord.

[128] Zigmond AS, Snaith RP (1983). The hospital anxiety and depression scale. Acta Psychiatr Scand.

[129] Giusti EM, Jonkman A, Manzoni GM (2020). Proposal for Improvement of the hospital anxiety and depression scale for the assessment of emotional distress in patients with chronic musculoskeletal pain: a bifactor and item response theory analysis. J Pain.

[130] Pilkonis PA, Choi SW, Reise SP (2011). Item banks for measuring emotional distress from the patient-reported outcomes measurement information system (PROMIS®): depression, anxiety, and anger. Assessment.

[131] Flens G, Smits N, Terwee CB et al (2017) Development of a computerized adaptive test for anxiety based on the Dutch-Flemish version of the PROMIS item bank. Assessment 1–13. 10.1177/1073191117746742 10.1177/1073191117746742 29231048

[132] Schalet BD, Pilkonis PA, Yu L (2016). Clinical validity of PROMIS depression, anxiety, and anger across diverse clinical samples. J Clin Epidemiol.

[133] de Castro NFC, de Melo Costa Pinto R, da Silva Mendonça TM, da Silva CHM (2020). Psychometric validation of PROMIS® Anxiety and Depression Item Banks for the Brazilian population. Qual Life Res.

[134] Klokgieters S, Mokkink LB, Galenkamp H, Beekman A, Comijs HC (2021). Use of CES-D among 56–66 year old people of Dutch, Moroccan and Turkish origin: measurement invariance and mean differences between the groups. Curr Psychol.

[135] Kroenke K, Spitzer RL (2002). The PHQ-9: a new depression diagnostic and severity measure. Psychiatr Ann.

[136] Vilagut G, Forero CG, Adroher ND, Olariu E, Cella D, Alonso J (2015). Testing the PROMIS(R) depression measures for monitoring depression in a clinical sample outside the US. J Psychiatr Res.

[137] Pilkonis PA, Yu L, Dodds NE, Johnston KL, Maihoefer CC, Lawrence SM (2014). Validation of the depression item bank from the Patient-Reported Outcomes Measurement Information System (PROMIS) in a three-month observational study. J Psychiatr Res.

[138] Jakob T, Nagl M, Gramm L, Heyduck K, Farin E, Glattacker M (2017). Psychometric properties of a German translation of the PROMIS® depression item bank. Eval Health Prof.

[139] Griggs S, Grey M, Ash GI, Li CR, Crawford SL, Hickman RL (2022). Objective sleep-wake characteristics are associated with diabetes symptoms in young adults with type 1 diabetes. Sci Diabetes Self Manag Care.

[140] Fabbri M, Beracci A, Martoni M, Meneo D, Tonetti L, Natale V (2021). Measuring subjective sleep quality: a review. Int J Environ Res Public Health.

[141] Savage CLG, Orth RD, Jacome AM, Bennett ME, Blanchard JJ (2021). Assessing the psychometric properties of the PROMIS sleep measures in persons with psychosis. Sleep.

[142] Chimenti RL, Rakel BA, Dailey DL (2021). Test-retest reliability and responsiveness of PROMIS sleep short forms within an RCT in women with fibromyalgia. Front Pain Res (Lausanne).

[143] Becker B, Raymond K, Hawkes C (2021). Qualitative and psychometric approaches to evaluate the PROMIS pain interference and sleep disturbance item banks for use in patients with rheumatoid arthritis. J Patient Rep Outcomes.

[144] Jones J, Nielson SA, Trout J (2021). A validation study of PROMIS Sleep Disturbance (PROMIS-SD) and Sleep Related Impairment (PROMIS-SRI) item banks in individuals with idiopathic Parkinson's disease and matched controls. J Parkinson's Dis.

[145] Donovan LM, Yu L, Bertisch SM, Buysse DJ, Rueschman M, Patel SR (2020). Responsiveness of patient-reported outcomes to treatment among patients with type 2 diabetes mellitus and OSA. Chest.

[146] Rose M, Bjorner JB, Gandek B, Bruce B, Fries JF, Ware JE (2014). The PROMIS physical function item bank was calibrated to a standardized metric and shown to improve measurement efficiency. J Clin Epidemiol.

[147] Abma IL, Butje BJD, Ten Klooster PM, van der Wees PJ (2021). Measurement properties of the Dutch-Flemish patient-reported outcomes measurement information system (PROMIS) physical function item bank and instruments: a systematic review. Health Qual Life Outcomes.

[148] Ziedas AC, Abed V, Swantek AJ (2022). Patient-Reported Outcomes Measurement Information System (PROMIS) physical function instruments compare favorably with legacy patient-reported outcome measures in upper- and lower-extremity orthopaedic patients: a systematic review of the literature. Arthroscopy.

[149] Zonjee VJ, Abma IL, de Mooij MJ (2022). The patient-reported outcomes measurement information systems (PROMIS®) physical function and its derivative measures in adults: a systematic review of content validity. Qual Life Res.

[150] Neijenhuijs KI, Holtmaat K, Aaronson NK (2019). The International Index of Erectile Function (IIEF)-a systematic review of measurement properties. J Sex Med.

[151] Neijenhuijs KI, Hooghiemstra N, Holtmaat K (2019). The Female Sexual Function Index (FSFI)-a systematic review of measurement properties. J Sex Med.

[152] Agochukwu NQ, Wittmann D, Boileau NR (2019). Validity of the Patient-Reported Outcome Measurement Information System (PROMIS) sexual interest and satisfaction measures in men following radical prostatectomy. J Clin Oncol.

[153] Reeve BB, Wang M, Weinfurt K, Flynn KE, Usinger DS, Chen RC (2018). Psychometric evaluation of PROMIS sexual function and satisfaction measures in a longitudinal population-based cohort of men with localized prostate cancer. J Sex Med.

[154] Weinfurt KP, Lin L, Bruner DW (2015). Development and initial validation of the PROMIS(®) sexual function and satisfaction measures version 2.0. J Sex Med.

[155] Iverson GL, Marsh JM, Connors EJ, Terry DP (2021). Normative reference values, reliability, and item-level symptom endorsement for the PROMIS® v2.0 cognitive function-short forms 4a, 6a and 8a. Arch Clin Neuropsychol.

[156] Valentine TR, Weiss DM, Jones JA, Andersen BL (2019). Construct validity of PROMIS® cognitive function in cancer patients and noncancer controls. Health Psychol.

[157] Noonan VK, Kopec JA, Noreau L, Singer J, Dvorak MF (2009). A review of participation instruments based on the International Classification of Functioning, Disability and Health. Disabil Rehabil.

[158] Hahn EA, Devellis RF, Bode RK (2010). Measuring social health in the patient-reported outcomes measurement information system (PROMIS): item bank development and testing. Qual Life Res.

[159] Terwee CB, Crins MHP, Boers M, de Vet HCW, Roorda LD (2019). Validation of two PROMIS item banks for measuring social participation in the Dutch general population. Qual Life Res.

[160] Fitzgerald JT, Davis WK, Connell CM, Hess GE, Funnell MM, Hiss RG (1996). Development and validation of the diabetes care profile. Eval Health Prof.

[161] Meadows KA, Abrams C, Sandbaek A (2000). Adaptation of the Diabetes Health Profile (DHP-1) for use with patients with Type 2 diabetes mellitus: psychometric evaluation and cross-cultural comparison. Diabet Med.

[162] Polonsky WH, Anderson BJ, Lohrer PA (1995). Assessment of diabetes-related distress. Diabetes Care.

[163] Sato E, Suzukamo Y, Miyashita M, Kazuma K (2004). Development of a diabetes diet-related quality-of-life scale. Diabetes Care.

[164] Goh SG, Rusli BN, Khalid BA (2015). Development and validation of the Asian Diabetes Quality of Life (AsianDQOL) Questionnaire. Diabetes Res Clin Pract.

[165] Orozco-Beltran D, Artola S, Jansa M, Lopez de la Torre-Casares M, Fuster E (2018). Impact of hypoglycemic episodes on health-related quality of life of type-2 diabetes mellitus patients: development and validation of a specific QoLHYPO((c)) questionnaire. Health Qual Life Outcomes.

[166] Mikhael EM, Hassali MA, Hussain SA, Shawky N (2020). The development and validation of quality of life scale for Iraqi patients with type 2 diabetes mellitus. J Pharm Bioallied Sci.

[167] Lin CY, Lee TY, Sun ZJ, Yang YC, Wu JS, Ou HT (2017). Development of diabetes-specific quality of life module to be in conjunction with the World Health Organization quality of life scale brief version (WHOQOL-BREF). Health Qual Life Outcomes.

[168] Huang Y, Wu M, Xing P (2014). Translation and validation of the Chinese Cardiff wound impact schedule. Int J Low Extrem Wounds.

[169] Hammond GS, Aoki TT (1992). Measurement of health status in diabetic patients. Diabetes impact measurement scales. Diabetes Care.

[170] Chuayruang K, Sriratanaban J, Hiransuthikul N, Suwanwalaikorn S (2015). Development of an instrument for patient-reported outcomes in Thai patients with type 2 diabetes mellitus (PRO-DM-Thai). Asian Biomedicine.

[171] Oobe M, Tanaka M, Fuchigami M, Sakata T (2007). Preparation of a quality of life (QOL) questionnaire for patients with type II diabetes and prospects for its clinical application. Fukuoka Igaku Zasshi.

