# Supplementary material for: Patient-reported outcomes for people with diabetes: what and how to measure? A narrative review
Source: Diabetologia. 2023 May 24;66(8):1357–77. doi: 10.1007/s00125-023-05926-3 (PMC10317894; doi:10.1007/s00125-023-05926-3)
Supplement: Supplementary file 1 — Supplementary file1 (PPTX 276 KB) [file 125_2023_5926_MOESM1_ESM.pptx]

## Slide 1
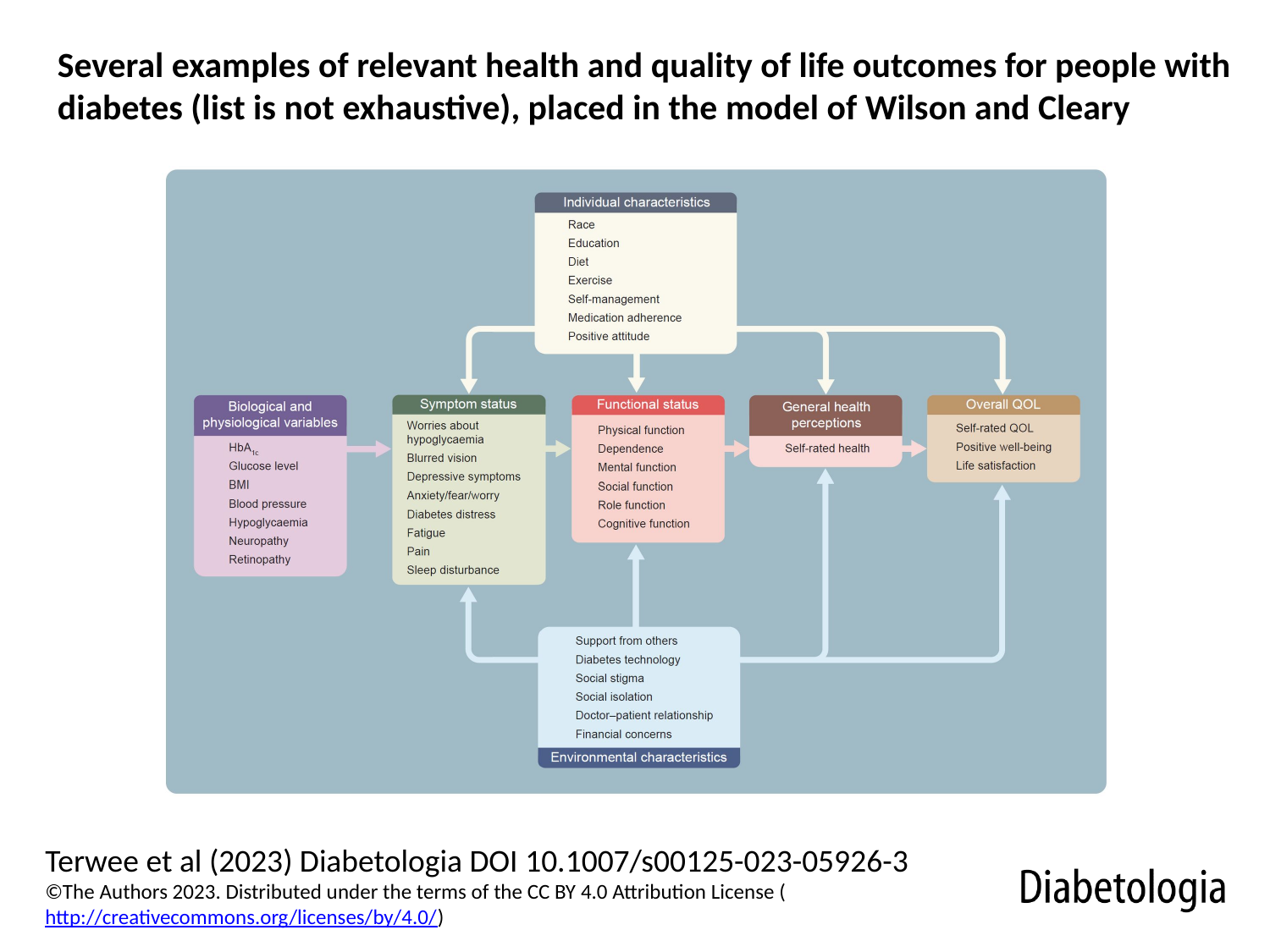

Several examples of relevant health and quality of life outcomes for people with diabetes (list is not exhaustive), placed in the model of Wilson and Cleary
Terwee et al (2023) Diabetologia DOI 10.1007/s00125-023-05926-3
©The Authors 2023. Distributed under the terms of the CC BY 4.0 Attribution License (http://creativecommons.org/licenses/by/4.0/)
